# The Effect of Single-Dose Administration of Dexamethasone on Postoperative Pain in Patients Undergoing Laparoscopic Cholecystectomy

**DOI:** 10.5812/aapm.17872

**Published:** 2014-08-13

**Authors:** Ahmadreza Mohtadi, Sholeh Nesioonpour, Amir Salari, Reza Akhondzadeh, Babak Masood Rad, Seyed Mohammad Mehdi Aslani

**Affiliations:** 1 Pain Research Center, Ahvaz Jundishapur University of Medical Sciences, Ahvaz, Iran; 2 Department of Anesthesiology, Ahvaz Jundishapur University of Medical Sciences, Ahvaz, Iran

**Keywords:** Laparoscopic Cholecystectomy, Postoperative Pain, Dexamethasone

## Abstract

**Background:**

Postoperative pain is considered as a reason of patient’s delay in discharge and disability aggravation. Therefore, multimodal approaches have been suggested in order to mitigate pain and decrease postoperative side effects.

**Objectives:**

The aim of this study was to evaluate analgesic effect of a single dose injection of dexamethasone on reducing postoperative pain in laparoscopic cholecystectomy.

**Patients and Methods:**

In this double-blind, prospective study, 122 patients aged 18-60 years old, whom were selected for laparoscopic cholecystectomy, were classified into two case and control groups, and 61 patients were included in each group. The case (D) group underwent general anesthesia and a single- dose intravenous injection of dexamethasone. The Control (C) group received general anesthesia and intravenous injection of normal saline. Total dose of consumed meperidine and pain intensity during first 24 hours were evaluated in both groups.

**Results:**

No significant difference existed between two groups regarding age, sex, weight and operation time. Pain intensity in group D was significantly less than group C (P < 0.01) after two, six and 12 hoursof surgery. No significant difference existed in pain intensity between two groups at the beginning of and 24 hours after the surgery (P > 0.05). Meperidine consumption in group D was significantly less than group C (P < 0.05).

**Conclusions:**

Findings of present study showed that single dose of intravenous dexamethasone, led to less pain intensity and amounts of meperidine consumption, in comparison with placebo.

## 1. Background

One the most important causes of patient’s illness after surgery is induced surgical trauma pain that can lead to chronic postoperative pain (1). Although, analgesic consumption after surgery can reduce postoperative side effects, but, pain is not controlled completely in most cases (2, 3). Laparoscopic cholecystectomy is a standard treatment for cholelithiasis, due to decreased postoperative trauma and its side effects. In spite of copious benefits of this approach, pain is still considered as most common complaint and the reason of prolonged hospitalization, increasing morbidity, and delayed functional recovery (4-6). Stretch of intra-abdominal organs, peritoneal inflammation and also phrenic nerve excitation by residual carbon dioxide (CO2) in peritoneal cavity are the causes of laparoscopic–induced pain (7).

Previous studies showed that preemptive analgesia causes attenuation of signals entering spinal cord, which is much more effective than controlling pain after its induction (8). Different treatments have been reported for pain relieving. Recently, multimodal analgesia approaches have been suggested to manage of postoperative pain. One of them is dexamethasone administration (9). Acute inflammation induced by tissue damage have a major role in development of postoperative pain, nausea and vomiting. Therefore, dexamethasone should be useful in lowering pain, nausea and vomitting, due to its potential anti- inflammatory effect. Dexamethasone is the most powerful anti-inflammatory drug with a long half- life; and its administration is considered safe for periods shorter than two weeks even in amounts above physiological doses (10, 11).

## 2. Objectives

The aim of this study was to investigate the effect of single- dose intravenous dexamethasone administration on postoperative pain in patients undergoing laparoscopic cholecystectomy.

## 3. Patients and Methods

This was a clinical double- blind, prospective study, carried out between 2012 and 2013. All of patients in case and control groups were selected from patients referred to Razi hospital in Ahvaz- Iran.

After proposal approval by ethical committee of Ahvaz Jundishapur University of Medical Sciences and obtaining informed consent and patients training by verbal explanation regarding how to assess pain intensity on visual analogue scale (VAS), 122 patients whom were candidate for laparoscopic cholecystectomy, aged 18-60 years, with American Society of Anesthesiologists (ASA) classes I and II were selected. Patients with hepatic and renal insufficiency, history of corticosteroid hypersensitivity, previous gastric ulcer, receiving corticosteroids or immunosuppressive drugs, diabetes mellitus, and receiving analgesics and opioids were excluded. Afterward, subjects were divided into two groups of 61 patients completely randomly with computer-generated list of random numbers.

After electrocardiographic monitoring, pulse oximetry, blood pressure measurement, capnography, and hydration with 10 mL/Kg of crystalloids in all subjects, general anesthesia was induced with midazolam (0.05 mg/Kg), fentanyl (2 µ/kg), sodium thiopental (5 mg/kg), and atracurium (0.5 mg/kg). Group D received 0.1 mg/kg (up to 8 mg) of dexamethasone, and group C received 2 ml of normal saline after anesthesia induction. At beginning of the surgery, four trocars were placed into the abdomen and surgical procedure initiated right after injection of CO_2_ and achieving intra-abdominal pressure of 14 mmHg. Patients received remifentanil (0.1 µ/kg/min) and propofol (50 µ/kg/min) as maintenance dose, to maintain anesthesia. Patients were reversed by neostigmine (0.05 mg/kg) and atropine (0.02 mg/kg). Both anesthesiologist and patients were not aware of performed classifications.

The post-operative pain intensity was measured with intervals of zero, two, six, 12 and 24 hours after entrance to postanesthesia care unit (PACU) by VAS, in which the minimum pain score is zero and the most severe pain that can be imagined is 10. A resident of anesthesiology, who was not aware of patient’s drug group, carried out these evaluations using a questionnaire. If pain score was equal or more than three, 0.2 mg/kg of meperidine was administered intravenously, and total consumed meperidine during 24 hours was recorded.

### 3.1. Statistical Analysis

Considering power of 80% and confidence interval (CI) of 95%, sample size was calculated as 61 samples for each group; therefore, 122 samples as a whole were included by NCSS software, based on variance analysis for repeated samples. All of the statistical data were reported as mean ± SD. After data collection, they were analyzed using SPSS version 18 software. To compare mean pain values in either case or control groups, independent samples t-test; and to compare pain scores at different hours square t-test and test of repeated measurements were applied. Significance of data was considered as P < 0.05.

## 4. Results

In the present study 122 patients were divided into two groups receiving placebo (C) and dexamethasone (D). There was no excluded patient. According to the results in Table 1, difference between two groups in terms of demographic characteristics (age, sex, weight and height) and duration of surgery was not significant; hence, random assignment is correct (P > 0.05).

**Table 1.** Demographic Characteristics and Duration of Surgery of Patients ^a,b^

	Saline group (n = 61)	Dexa group (n = 61)	P Value
**Age, y**	47.26 ± 5.93	46.7 ± 5.78	0.600
**Gender**			0.911
Female	46	47	
Male	15	14	
**Weight, kg**	64.9 ± 6.1	65.5 ± 6.42	0.629
**Height, cm**	162 ± 5.75	162 ± 5.45	0.963
**Duration of surgery, min**	63.5 ± 9.65	63.8 ± 10.3	0.898

^a^ Data are presented as numbers or mean ± SD.

^b^ P < 0.05 shows significant difference between groups.

According to the results in Table 2, total consumed postoperative meperidine, in intravenous dexamethasone receiving group was significantly less than placebo receiving group (P = 0.03). According to Table 3, mean postoperative pain intensity based on VAS score at two, six and 12 hours after entrance to PACU in dexamethasone receiving group was significantly lower compared with placebo group (P < 0.05). Based on VAS pain score, mean postoperative pain intensity at zero and 24 hours after entrance to PACU was not significantly different in groups receiving dexamethasone and placebo.

**Table 2.** Total Analgesic Consumption ^a^

	Saline group (n = 61)	Dexa group (n = 61)	P Value
**Total I.V. Meperidine consumption, mg**	60.24 ± 10.55	44.26 ± 7.32	0.03 ^b^

^a^ Data are presented as numbers or mean ± SD.

^b^ significant difference between groups (P < 0.05).

**Table 3.** Pain Intensity (VAS) ^a^

VAS	Saline group (n = 61)	Dexa group (n = 61)	P Value
**On arrival to PACU (0)**	5.29 ± 2.88	5.21 ± 2.88	0.875
**After 2 hours**	5.15 ± 2.80	3.88 ± 1.03	0.010 ^b^
**After 6 hours**	4.86 ± 2.78	3.08 ± 1.79	0.020 ^b^
**After 12 hours**	4.03 ± 1.94	3.03 ± 1.74	0.003 ^b^
**After 24 hours**	3.11 ± 1.79	2.95 ± 1.85	0.621

^a^ Data are presented as numbers or mean ± SD.

^b^ significant difference between groups (P < 0.05).

## 5. Discussion

Results of present study revealed that intravenous single-dose of dexamethasone can reduce postoperative pain at first 12 hours in comparison with placebo. The intravenous single-dose dexamethasone can also decrease total consumed analgesic. Lim et al. found that intravenous injection of dexamethasone before and after laparoscopic cholecystectomy has been effective in reducing postoperative pain (9). Also, study carried out by Fukami et al. showed that 8 mg of intravenous dexamethasone has significantly reduced postoperative pain and fatigue after laparoscopic cholecystectomy (12), which is similar to our study.

Nevertheless, Elhakim et al. showed that while the intravenous dexamethasone was effective in reducing postoperative nausea and vomiting, it was not effective in case of postoperative pain (13). Pain after laparoscopic cholecystectomy could be induced by skin incision, visceral pain and shoulder pain due to diaphragmatic irritation. Furthermore, multiple factors such as different individual characteristics, nature of underlying diseases, surgical factors and type and volume of gas and also induced intra-abdominal pressure are effective on postoperative pain (6, 9, 14).

Since multiple factors are considered for postoperative pain, application of approaches that leads to short- term analgesia cannot cause functional improvement and reduction of the length of hospitalization; hence, the multimodal approaches are considered (6, 9). Because of advanced surgical procedures and modern anesthesia, 84% of patients are discharged during the first 24 hours after laparoscopic cholecystectomy. Administration of steroids is effective in reducing postoperative pain, mood improvement, reducing fatigue and increasing appetite after operation (15).

 Strong anti-inflammatory properties of dexamethasone have caused to introduction of “dexamethasone induced postoperative pain reduction” theory. Although analgesic mechanism of dexamethasone is still unclear, it seems that a decrease in cyclooxygenase and lipoxygenase production, via inhibition of peripheral phospholipase, plays a main role (16, 17).

While long- term consumption of steroids is associated with some side effects such as increased risk of wound infection, delayed wound healing, adrenal suppression and glucose intolerance, their single-dose administration is safe (11, 13). Performed meta- analyses about effect of dexamethasone on postoperative pain revealed that more dexamethasone (base on mg/kg) is accompanied with higher effectiveness. According to performed investigations, dexamethasone receiving patients had a higher blood glucose level during the first 24 hours after injection (18, 19).

The study showed that time of dexamethasone injection is important on reducing postoperative pain as well; since initiation of its biological effect is one to two hour after injection (15). In our study, it might be one of reasons for insignificant VAS score difference between two groups at the time of entrance recovery. One of the limitations of the present study was following-up patients just in 24 hours after operation. Study of different dexamethasone doses at different times and also measurement of serum concentration of drug and stress hormones could be performed in other researches.

Laparoscopic cholecystectomy is one of selective surgical approaches for cholecystitis, which is done frequently. Therefore, achieving sufficient analgesia for this procedure is very important. Regarding the present study, it seems that intravenous single-dose dexamethasone can reduce postoperative pain and opioid consumption.
